# Postoperative Closed-Suction Drain in Anterior Orbitotomy

**DOI:** 10.7759/cureus.48624

**Published:** 2023-11-10

**Authors:** Pankaj Gupta, Manu Saini, Vijay K Sharma, Manpreet Singh, Khushdeep Abhaypal, Aditi Mehta

**Affiliations:** 1 Ophthalmology, Postgraduate Institute of Medical Education and Research, Chandigarh, IND

**Keywords:** post-operative oedema, closed suction, proptosis, orbital drain, anterior orbitotomy

## Abstract

Purpose

To evaluate the role of a closed-suction drain in orbital mass excision following anterior orbitotomy.

Methods

This is a prospective, randomized comparative study of consecutive patients undergoing anterior orbitotomy and mass excision enrolled into two groups: group A (with drain) and group B (without drain). Clinical data included visual acuity assessment, proptosis measured by exophthalmometry, pain score assessment, eyelid swelling, and ocular motility. Postoperative data were compared for one to five days and at 14 and 30-day follow-ups in the two groups to evaluate the efficacy of closed-suction drain in orbital mass excision.

Results

Twenty-five patients planned for anterior orbitotomy were divided into two groups: group A (drain, n = 12) and group B (without drain, n = 13). The subsidence of proptosis (p = 0.041), eyelid swelling (p = 0.04), and restoration of ocular motility (p = 0.04) were faster in the drain group as compared to the non-drain group, which was observed as statistically significant. The outcomes at 30 days were comparable in both groups and none of the patients developed any long-term complications.

Conclusion

The use of orbital drains aids early postoperative recovery with faster subsidence of proptosis and eyelid edema, and rapid recovery of ocular movements but does not affect the final outcome. Orbital surgeons can individualize the use of closed-suction drains after anterior orbitotomy in cases with expected postoperative edema.

## Introduction

The orbit is a pyramid-shaped bony socket, narrowed posteriorly at the apex with a quadrangular base at the anterior aspect. It is divided into numerous surgical spaces enumerated as intraconal, extraconal, subperiosteal, and sub-tenon spaces. Orbital mass, according to their location, exerts a pressure effect resulting in proptosis, globe displacement, restriction of ocular movements, optic neuropathy resultant loss of vision, and prominent esthetic concerns. Moreover, orbital masses can pose a life-threatening risk if malignant. Surgical excision via orbitotomy is the preferred choice of management for a well-localized orbital mass. Anterior orbitotomy is defined as a trans-cutaneous or trans-conjunctival approach that may or may not involve the removal of a bony orbital wall devised by Knapp [1] and popularized by Benedict [2]. An anterior orbital lesion located in the anterior two-thirds compartment of the orbit is amenable for excision via this approach [1]. Surgical excision of masses leaves behind a potential space for fluid/blood collection that may inhibit wound healing and potentiate the risk for infection [3]. Orbital hemorrhage in this space can lead to compartment syndrome either during or after surgery with as little as 7 ml fluid accumulation [4]. The source of hemorrhage can be from inadequate cauterization of bleeders in the operative field or laceration of ethmoidal vessels along the medial orbital wall. In a retrospective study by Jacobs et al. [5], the overall incidence of severe loss of visual acuity after orbital surgery was reported to be 0.84%.

Placement of a drain in the cavity has been shown to reduce the morbidity and complications following abdominal, plastic, and orthopedic surgery [6-8]. Somers et al. (1992) reported that postoperative closed-suction drainage is advantageous in decreasing the incidence and degree of seroma formation after lumpectomy and axillary lymph node dissection. The authors also mentioned "no delay" in getting discharged from the hospital [9]. Therefore, two types of drainage were utilized during the surgery, namely, active drain and passive drain. They are further categorized into either open or closed drain systems [10]. Closed-suction drains after orbitotomy facilitate in removing collecting fluid, thereby mitigating the “dead space” [11]. However, they are not without potential risks. The frequent occurring complications associated with the placement of a drain in a closed cavity include hemorrhage, inflammation, retrograde bacterial migration, loss or entrapment of the drain, pain, and possible protein-electrolyte loss [12]. Therefore, there may be concern about its placement and management dexterity that evidently led to a debate on the role of the postoperative drain. On reviewing the literature, there is a dearth of decisive evidence on the utility of closed-suction drain in orbitotomy; however, few authors have recommended it in all facial and orbital fracture repairs [4,13,14]. Hence, the study was designed to compare the outcome and efficacy of anterior orbitotomy with or without closed suction drains.

## Materials and methods

This study was designed as a prospective, randomized comparative study of consecutive patients undergoing anterior orbitotomy over 15 months. After obtaining written informed consent, adult patients with anterior orbital mass, requiring excision by anterior orbitotomy were randomized into two groups, i.e., with (group A) and without (group B) postsurgical closed-suction drains. Randomization was performed using a random number table. The study was conducted according to the tenets of the Declaration of Helsinki. Patients with known bleeding disorders, uncontrolled systemic hypertension or diabetes, and a deranged coagulation profile were excluded. The Institutional Ethics Committee approval was sought and obtained (Postgraduate Institute of Medical Education and Research, Chandigarh; Approval Number: NK/2177/MS/10753-54). The preoperative ophthalmic examination comprised best-corrected visual acuity assessment, exophthalmometry measurement, ocular movement limitation assessment, description of a palpable orbital mass, and anterior and dilated posterior segment examination. Clinical photography of patients’ faces was done using a digital camera and consent to publish identifiable photographs in our study was obtained. Systemic investigations included a complete hemogram, blood sugar levels, blood pressure measurement, coagulation profile, and renal function tests. Imaging of the orbital mass was done using computed tomography (CT) scan orbit modality in all the patients.

Surgical technique

The patients underwent anterior orbitotomy with mass excision using the standard of care under general anesthesia. Intraoperatively, globe manipulation was kept to a minimum, and periodic evaluation of pupillary reaction was performed to guide the dissection and avoid inadvertent damage to the optic nerve. Minimal cautery and adrenaline-soaked gauze were used for hemostasis. All patients were operated upon by either PG or ZZ. The Mini Vac closed suction drain (Romsons Scientific and Surgical Pvt. Ltd., Agra, India, size: 8 French, 2.70 mm) was placed in group A enrolled patients. The drain was kept in the deep of the wounds, brought out through a separate incision in the skin, and sutured to the skin to prevent slippage with airtight wound closure. This was left in place until the collection was less than 5 ml in 24 hours. The wound was inspected daily for hematoma, oozing, and infection. The time from operation to the removal of the drain and the total volume collected in the drain were recorded. The patients were discharged after the removal of the drain.

The patients in group B underwent the orbitotomy with the same standard of care. However, no drain was placed. Postoperatively, patients were followed up regularly for five days on an OPD basis to observe any inadvertent reaction.

Pain assessment

A visual analog scale (VAS) was used to calculate pain on a continuous scale ranging from 0 mm to 100 mm on a horizontal straight line [15]. The patients were asked to point on the ruler the level of pain perceived by them. The horizontal straight-line ruler ranged from no pain to mild, moderate, and severe pain. The cut points recommended were no pain (0-4 mm), mild pain (5-44 mm), moderate pain (45-74 mm), and severe pain (75-100 mm) [16].

Next, all patients were evaluated postoperatively for five consecutive days and at days 14 and 30 to assess visual acuity, proptosis by Hertel’s exophthalmometry, ocular movements limitations, presence of subconjunctival hemorrhage, eyelid and/or periocular swelling, and ecchymosis. The ocular motility limitation was determined by clinical assessment of eye movements within the known field of extraocular muscle activity and resulting diplopia was evaluated on the basis of subjective reporting using red and green glasses.

Eyelid swelling was measured using a four-grade scale method developed by Gurlek et al. [17], ranging from phases 0 to 4, where phase 1 encompassed minimal edema, phase 2 represents open eyelids reaching the iris due to the effect of edema, phase 3 involved open eyelids showing only the pupils due to the effect of edema, and phase 4 comprised eyelids completely closed due to generalized edema. Ecchymosis [18] was assessed on a slit lamp examination graded from nil to mild, moderate, and severe, resulting in a score between 0 and 3, where mild being just visible, moderate denotes fairly obvious, and severe represents protruding out.

A torchlight examination was performed for suture site evaluation, measurement of fluid collection in the drain, drain-related complications, drain breakage, undue difficulty in drain removal, infection at the drain site, occlusion of the drain lumen, and pain at the drain site. The mean time to resolution of proptosis, ocular motility, eyelid swelling, change in visual acuity, postoperative complications, and histopathology of orbital masses was noted for both groups.

Statistical analysis

Statistical analysis was performed using SPSS software (IBM Corp., Armonk, NY). Qualitative variables were expressed as mean ± standard deviation. Two-tailed t-tests were used to compare visual acuity, eyelid swelling, extraocular muscle restriction, and pain assessment in the two studied groups and a p-value less than 0.05 was considered statistically significant.

## Results

A total of 25 patients were enrolled in the study. Of these, the majority (16 patients, 64%) were females. The mean age at presentation was 43.56 ± 15.77 years. Twelve patients were allocated to group A and 13 to group B. The principal ocular complaint was a forward protrusion of the eye (88%), followed by a restriction of ocular movements (84%). The preoperative mean visual acuity of the affected eye in group A was 0.08 ± 0.20 and in group B was 0.11 ± 0.18 (p = 0.39) and remained consistent after the surgery. The demographic details are summarized in Table 1. All orbital masses were approached via anterior orbitotomy, superior or inferior depending upon the localization of orbital mass, as summarized in Table 1. The mean surgical time was 75 ± 15 minutes. The time taken for drain placement was less than five minutes in group A patients. The surgical procedure remained uneventful and none of the patients developed any intraoperative complications.

**Table 1:** Demographic details and clinical data of the recruited patients in group A (with drain) and group B (without drain). OD: right eye; OS: left eye.

Characteristics	Group A (n = 12)	Group B (n = 13)	p-value
Age (years)	44.67 ± 1 6.35	42.54 ± 15.8	0.21
Male:female	4:8	5:8	0.71
Chief ocular complaint			
Swelling of eyelid	11 (91.7%)	10 (76.9%)	1.009
Restricted motility	10 (83.3%)	11 (84.6%)	1.0
Protrusion of eye	10 (83.3%)	12 (92.3%)	0.476
Decreased vision	1 (8.3%)	4 (30.8%)	1.963
Headache	3 (25%)	2 (15.4%)	0.361
Double vision	2 (16.7%)	1 (7.7%)	0.476
Watering of eye	2 (16.7%)	1 (7.7%)	0.476
Laterality			
OD:OS	6:6	6:7	0.37
Mean visual acuity (log MAR)			
Preoperative	0.08 ± 0.20	0.11 ± 0.18	0.39
Location			
Superotemporal	4 (33.3%)	3 (23.0%)	
Superonasal	5 (41.6%)	5 (38.46%)	
Inferonasal	1 (8.3%)	2 (15.3%)	
Inferotemporal	1 (8.3%)	2 (15.3%)	
Inferior	1 (8.3%)	1 (7.6%)	
Surgical approach			
Superior transconjunctival	11 (91.6%)	9 (69.3%)	
Inferior transconjunctival	1 (8.3%)	4 (30.7%)	

In group A (drain group), the mean collection of fluid was 8 ± 2.63 ml on postoperative day one, 4.83 ± 1.403 ml on postoperative day two, and 1 ± 2.0 ml on day three. The closed suction drain was removed once the collection was less than 5 ml in 24 hours using the standard procedure of drain removal. In four (33.3%) cases, it was removed at 24 hours, in seven patients (58.3%) at 48 hours, and in one (8.3%) case, it was removed 72 hours after the surgery. The postoperative clinical parameters evaluated in the enrolled groups A and B at serial follow-up are summarized in Table 2.

**Table 2:** Comparison of clinical outcomes between group A (with drain) and group B (without drain). * Statistically significant value; VAS: visual analog scale.

Characteristics	Group A (n = 12)	Group B (n = 13)	p-value
Mean visual acuity in Log MAR			
Preoperative	0.08 ± 0.20	0.11 ± 0.18	0.39
Postoperative	0.08 ± 0.20	0.11 ± 0.18	0.39
Proptosis evaluation			
Preoperative	10	12	0.476
Day 1	3 (25%)	9 (69.2%)	0.047*
Day 2	3 (25%)	9 (69.2%)	0.047*
Day 3	2 (16.6%)	8 (61.5%)	0.047*
Day 4	2 (16.6%)	8 (61.5%)	0.041*
Day 5	1 (8.3%)	7 (53.8%)	0.041*
Day 14	1 (8.3%)	3 (23%)	0.59
Day 30	0	0	-
Restriction of ocular movement			
Preoperative	10	11	1
Day 1	9	11	0.64
Day 2	6	11	0.09
Day 3	4	11	0.015*
Day 4	0	8	0.001*
Day 5	0	5	0.03*
Eyelid swelling			
Preoperative	11	10	1.009
Day 1	12	13	0
Day 5	2	8	0.04*
Day 14	0	0	1
Day 30	0	0	1
Pain assessment on VAS			
Day 1	1.92 ± 1.2	3.083 ± 0.66	0.023
Day 2	1.92 ± 1.2	3.083 ± 0.66	0.023
Day 3	1.38 ± 1.2	2.66 ± 0.88	
Day 4	1.38 ± 1.2	2.66 ± 0.88	
Day 5	1.47 ± 1.2	2.08 ± 0.9	
Postoperative complications			
Wound infection	0	0	
Sub-conjunctival hemorrhage	12	13	
Ecchymosis	3	6	0.41
Ptosis	3	6	0.41
Diplopia	0	1	0.96
Deterioration of vision	0	0	
Deviation of eye drain removal (postoperative hours)	141.45 ± 14.28	0	
Visible wound scar	4	3	

The subsidence of proptosis (p = 0.04), eyelid swelling (p = 0.03), and restoration of ocular motility (p = 0.04) were faster in the drain group as compared to the non-drain group, which was observed statistically significant on day five. On comparing the VAS scores for pain in the two groups, a statistically significant (p = 0.023) difference was observed, and postoperative pain continued in group B on day five as compared with group A.

None of the patients developed wound infection or deterioration of vision in either group. All patients developed subconjunctival hemorrhage. Three patients (25%) in group A and six (46.15%) in group B developed ecchymosis of the lid and mechanical ptosis as an early complication. One of the group B patients developed diplopia in the early postoperative period, which was resolved on follow-up. In group A, four patients had visible wound scars, while in group B, one patient had ptosis and three patients had visible wound scars.

Histopathology examination of the excised orbital masses in group A disclosed pleomorphic adenoma of the lacrimal gland (n = 5), cavernous hemangioma (n = 3), lymphoma (n = 1), non-specific orbital inflammatory lesion (n = 1), orbital dermoid cyst (n = 1), and arteriovenous malformation (n = 1).

Group B patients confirmed orbital lymphoma (n = 1), benign orbital cyst (n = 4), schwannoma (n = 5), cavernous hemangioma (n = 2), and lipoma (n = 1) on histopathological examination.

The orbital masses were removed in toto for all the lesions except lymphoma, non-specific orbital inflammatory lesion in group A, and orbital lymphoma in group B, where near total excision was performed.

## Discussion

The surgical drain usage in orbital vascular malformations primarily high flow communication seems prudent, involving excision or debulking of vascular lesions; however, their utility in non-vascular or relatively low vascular orbital masses is dubious. Their beneficial role in thoracic and abdominal surgeries [19,20] has been extensively described. The use of drains has been related to the increased duration of hospital stay in abdominal and orthopedic surgeries [21,22]. Therefore, the above notions inspire us to study the safety and efficacy of postoperative orbital drain in anterior orbitotomy.

There are studies in the literature that advocate the favorable outcome of the postoperative orbital drain. Purgason et al. [23] recommended the placement of postoperative orbital drains in all cases of orbitotomy for a minimum period of 48 hours. Another study of a sample size of 92 patients undergoing orbital fracture repair demonstrated the reduced pooling of blood in the maxillary sinus following drain placement [14]. The authors also shared their non-randomized, retrospective review of 86 orbitotomy with an earlier resolution of postoperative edema following orbital drain [21]. However, in the present scenario, the use of drains in orbitotomy is dictated by the surgeon’s preference and restrained by its undue side effects. Hence, the study is exclusive as it compares the postoperative resolution of clinical parameters in the two groups with and without drain.

Our study documented no significant difference in the visual acuity following postoperative closed suction drain in the two groups and observed statistically comparable in the pre and postoperative periods (p = 0.39). None of the patients reported any transient or persistent vision loss in the postoperative period. However, Singh and co-workers [24] reported visual function improvement following orbital mass excision (p = 0.002). Their recuperation was attributed to the resolution of posterior segment changes, namely, disc pallor, disc edema, and choroidal folds, and correlated with the location, type, and extent of the mass. As the anterior two-thirds located orbital masses were recruited for anterior orbitotomy, rather than posterior squashed space impinging on the optic nerve, perhaps explain no significant vision improvement in our study groups.

The subsidence of proptosis (p = 0.041), eyelid swelling (p = 0.04), restoration of ocular motility (p = 0.03), and pain resolution (p = 0.023) observed on day five postoperative follow-up were noted as statistically significantly faster in the drain group. Anecdotally, most drainage occurs in the early postoperative hours and declines considerably after this period. The presence of a drain during this period encourages the drainage of accumulating fluid in postoperative created dead space by stimulating tissue reactions or by creating a suction effect [25] and therefore resulted in expedited recovery in group A patients. Moreover, there was an apparent early reduction in eyelid ecchymosis and ptosis in the drain group as compared to the non-drain group, though it was not perceived as significant (p = 0.41). However, interestingly, at one-month follow-up, clinical parameters, i.e., proptosis, ocular motility, and pain perception, were comparable and normalized in the two study groups.

The mean collection of fluid in the drain was less than 10 ml in the follow-up period and was removed 72 hours after surgery in all the group A recruited patients. Surgical duration, hemostatic agents, and intraoperative bleeders are pertinent to the expected accumulation of fluid in the immediate postoperative dissected tissue spaces. However, in our study, no enteral or parenteral administration of hemostatic agents was used. The anterior orbitotomy surgeries were completed in a rational time duration with gentle dissection and complete excision of the tumor, possibly explaining the concordant results in the two groups, notably at one-month follow-up.

The presence of a drain, therefore, did not prolong the hospital stay for any patient. This is in contrast to studies on laparoscopic cholecystectomy and thyroid surgery associated with extended duration of hospital stay and greater chances of infection in the drain group [17,18]. Notably, none of the patients in either group developed any orbital compartment syndrome.

Therefore, the use of orbital drains aids the early postoperative recovery with faster subsidence of proptosis, eyelid edema, and rapid recovery of ocular movements; however, the outcome was comparable in both groups. The main limitation of our study is the small sample size. The type of orbitotomy was limited to the anterior approach for lesions in the anterior two-thirds of the orbit. A larger study with a wider sample size would perhaps help in determining the role of prophylactic drains for faster resolution and mitigation of postoperative orbital compartment syndrome and vision loss.

## Conclusions

Our study highlights the role of the postoperative drain in anterior orbitotomy and emphasizes that drain may be considered in anterior orbital lesions having a high propensity of bleeding and postoperative compartment syndrome to expedite resolution in proptosis, ecchymosis, restoration of ocular motility, and alleviation in pain. However, the final clinical outcomes were indiscernible in the drain and without the drain groups.

## Figures and Tables

**Figure 1:**
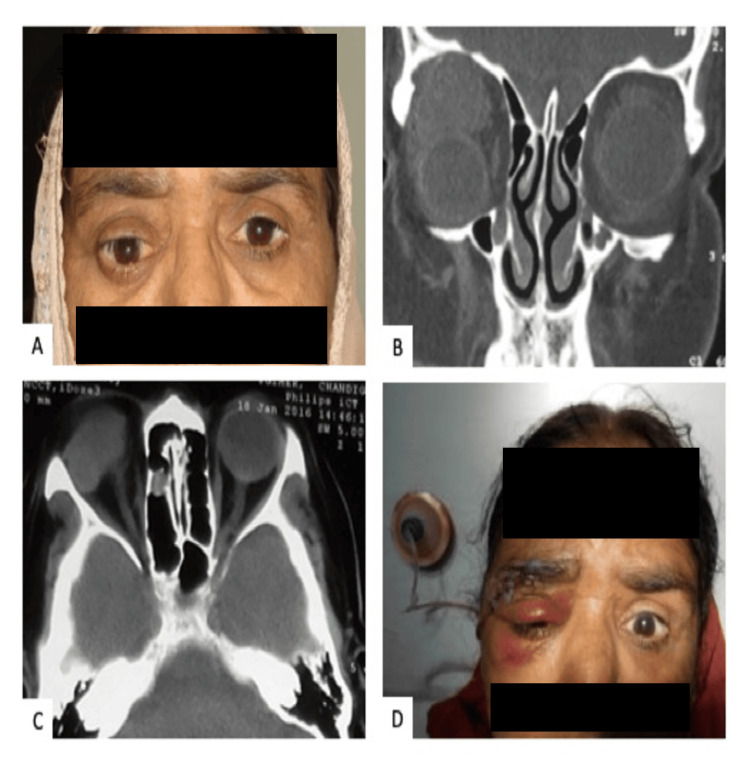
(A) Middle-aged female patient of study group B with fullness and proptosis of the right eye. (B and C) A CT scan shows an orbital mass in the superonasal quadrant and anterior two-thirds of the orbit. (D) On postoperative day one, proptosis, ecchymosis of the lid, and ptosis were present.
